# Recurso público na agenda brasileira da Segurança Alimentar e Nutricional de 2000 a 2022

**DOI:** 10.11606/s1518-8787.2024058006104

**Published:** 2024-12-20

**Authors:** Milena Corrêa Martins, Mick Lennon Machado, Patrícia de Fragas Hinnig, Patrícia Maria de Oliveira Machado, Cristine Garcia Gabriel

**Affiliations:** I Universidade Federal de Santa Catarina. Centro de Ciências da Saúde. Programa de Pós-Graduação em Nutrição. Florianópolis, Santa Catarina, Brasil; II Universidade Federal de Santa Catarina. Centro de Ciências da Saúde. Teia de Articulação pelo Fortalecimento da Segurança Alimentar e Nutricional (TearSAN). Florianópolis, Santa Catarina, Brasil; III Universidade de Brasília. Faculdade de Ciências da Saúde. Departamento de Nutrição. Brasília, Distrito Federal, Brasil; IV Universidade Federal de Santa Catarina. Centro de Ciências da Saúde. Programa de Pós-Graduação em Saúde Coletiva. Florianópolis, Santa Catarina, Brasil

**Keywords:** Recurso Público, Segurança Alimentar e Nutricional, Estudos de Séries Temporais

## Abstract

Analisar a tendência temporal dos recursos públicos federais investidos na agenda brasileira de Segurança Alimentar e Nutricional do período de 2000–2022.

Realizou-se um estudo de série temporal com dados coletados de um sistema de acesso público sobre planejamento e orçamento do Governo Federal. Foram analisadas as ações orçamentárias e seus recursos indicados pela Dotação Orçamentária e o Valor Liquidado. As ações foram categorizadas em temas da agenda de SAN, analisadas a partir da regressão de Prais-Winsten, juntamente com o método de Cochrane-Orcutt. Os valores foram corrigidos pelo Índice de Preços ao Consumidor Amplo, para janeiro de 2023.

O investimento na área de Segurança Alimentar e Nutricional apresentou uma tendência crescente em duas décadas de financiamento federal, apesar de ter demonstrado oscilações em determinados anos. A Dotação Orçamentária mostrou uma variação anual de 10,1%, e o Valor Liquidado obteve 10,8%. No entanto, há um desequilíbrio no financiamento federal entre as áreas estratégicas, com tendência crescente na saúde (37,4%), soberania alimentar (23,7%) e acesso à alimentação (12,3%); e tendência negativa de investimento em algumas áreas, como desenvolvimento agrário (-7,0%). Constatou-se uma alocação de recursos concentrada em ações assistenciais para garantia do acesso à alimentação, em média 73,4% do total de recursos executados.

Os resultados apresentaram um comportamento crescente do investimento público na agenda brasileira de Segurança Alimentar e Nutricional, embora expressassem oscilações em determinados anos e uma concentração de recursos em áreas específicas. Apesar de 2/3 do orçamento ser exclusivamente para ações de acesso à alimentação, dados recentes demonstram que a fome ainda assola a realidade brasileira. Reforçando a necessidade de um investimento contínuo e mais equânime entre as áreas, como forma de fortalecer políticas públicas estruturantes que garantam de forma permanente a Segurança Alimentar e Nutricional da população brasileira.

## INTRODUÇÃO

 A insegurança alimentar (IA) mundial tem aumentado progressivamente, com cerca de 900 milhões de pessoas em situação de IA Grave em 2022 ^1^ . Projeções do International Food Policy Research Institute indicam que 65 milhões de pessoas a mais estarão nesta situação até 2030 ^2^ . Na mesma direção dos dados globais, 33,1 milhões de pessoas estavam em IA grave no Brasil do período de 2021 a 2022 ^3^ . 

 Esse cenário tem mobilizado diferentes países e organizações para formulação e destinação de recursos para a agenda de Segurança Alimentar e Nutricional (SAN) ^4^ . Destacam-se algumas estratégias nacionais, geralmente de caráter intersetorial, com alocação de recursos em diversas áreas de SAN, tais quais: saúde, educação, proteção social, agricultura, meio ambiente, entre outros ^5^^,^^6^ . Ao longo da pandemia de covid-19, tais estratégias contaram com a ampliação dos recursos, diante do aumento da IA ^7^^,^^8^ . 

 As primeiras medidas adotadas por 54 países em resposta à pandemia, envolveram 496 ações e um investimento público de US$ 47,6 bilhões relacionados à SAN ^9^ . Em 2022, o Banco Mundial como parte de uma resposta global à crise de IA, disponibilizou 30 milhões de dólares para o financiamento de projetos relacionados à área ^10^ . 

 O Brasil intensificou suas ações de enfrentamento à fome a partir do início da década de 2000, com destaque para a publicação da Lei Orgânica de Segurança Alimentar e Nutricional (Losan) em 2006, que institucionaliza no país uma agenda pública organizada intersetorialmente por meio do Sistema Nacional de Segurança Alimentar e Nutricional (Sisan), responsável por retirar o Brasil do Mapa da Fome em 2014 ^11^ . De acordo com o Hunger And Nutrition Commitment Index (HANCI), um índice mundial que avalia o compromisso político nacional para combater a fome e a IA considerando variáveis financeiras, em 2019 o Brasil ocupou o ranking de segundo lugar dentre quarenta e cinco países de renda média-baixa analisados ^12^ . 

 Na lógica institucional brasileira o orçamento público é o instrumento utilizado para planejar a utilização do dinheiro arrecadado com os tributos. A sua análise possibilita a compreensão das escolhas governamentais e da trajetória das políticas, revelando o quanto se gasta e as agendas prioritárias. Neste sentido, análises que possibilitem comparações ao longo do tempo subsidiam informações sobre as características das agendas de governo, as pautas priorizadas e as ações implantadas para executar a agenda de SAN ^13^ . Considerando a escassez de pesquisas na área, este estudo buscou analisar a tendência temporal dos recursos públicos federais investidos na agenda brasileira de SAN ao longo das duas últimas décadas (2000 e 2022), período marcante por avanços significativos, mas também por retrocessos substanciais na área. 

## MÉTODOS

 Trata-se de estudo de série temporal do montante de recursos públicos federais investidos na área de SAN no período de 2000 a 2022. Os dados foram coletados no Sistema Integrado de Planejamento e Orçamento (SIOP) ( https://www.siop.planejamento.gov.br ), um sistema de acesso público que reúne as informações sobre planejamento e orçamento do Governo Federal. Foram analisadas as ações orçamentárias, e seus recursos indicados pela dotação orçamentária e o valor liquidado. A dotação orçamentária diz respeito ao valor previsto e aprovado no orçamento público, enquanto o valor liquidado retrata o que efetivamente foi executado no orçamento. 

Dada a complexidade e a multidimensionalidade da agenda de SAN, este estudo enfrentou o desafio metodológico de determinar quais ações orçamentárias seriam incluídas na análise. Optou-se por critérios de inclusão mais amplos, garantindo assim a análise de ações orçamentárias associadas às diretrizes da Política Nacional de Segurança Alimentar e Nutricional (PNSAN), sem se restringir exclusivamente àquelas vinculadas ou originadas diretamente dessa política pública. Essa abordagem teórico-metodológica baseia-se na compreensão de que a natureza intersetorial da PNSAN implica na alocação e utilização de recursos públicos ligados a diferentes políticas para a efetiva concretização da agenda de SAN no país. Essa compreensão também é refletida no principal instrumento de planejamento e gestão da PNSAN, os Planos Nacionais de SAN, que descrevem ações orçamentárias vinculadas a diversas políticas para o cumprimento de suas metas.

**Figura 1.**
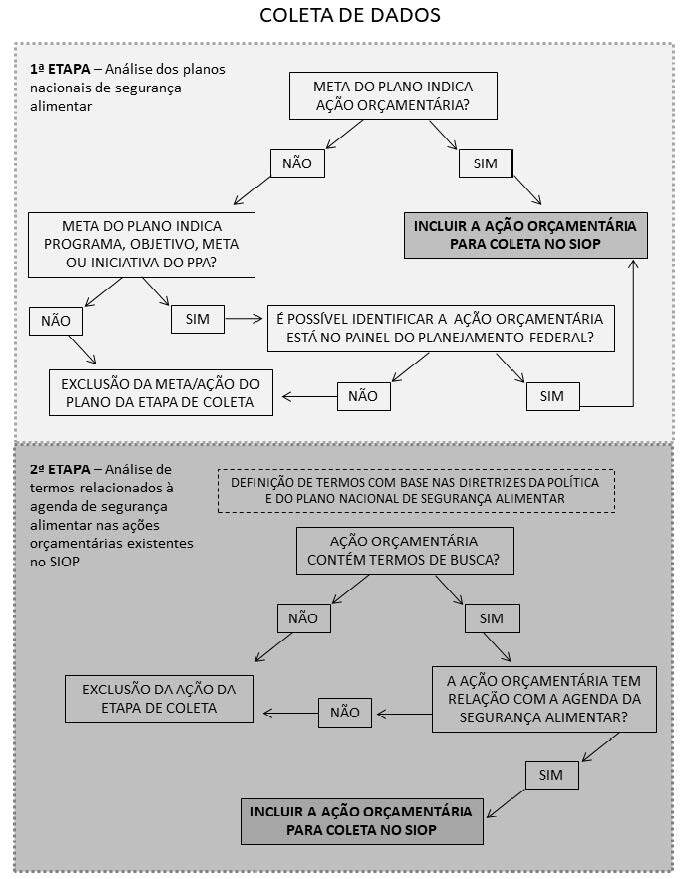
Protocolo de identificação das ações orçamentárias relacionadas à agenda de Segurança Alimentar e Nutricional no Sistema Integrado de Planejamento e Orçamento. Brasil, 2023.

 Por isso, a identificação das ações orçamentárias associadas à agenda de SAN foi realizada em duas etapas, com base em um protocolo previamente construído e pactuado entre os pesquisadores ( Figura 1 ). Inicialmente, as ações orçamentárias foram definidas com base nas metas dos Planos Nacionais de SAN. Foram analisados quatro documentos: a primeira versão do plano (período de 2012–2015), a segunda versão (período 2016–2019), bem como, suas respectivas revisões. Como estes documentos apresentam as ações orçamentárias de forma diferente, estabeleceu-se um fluxograma para a padronização da coleta de dados ( Figura 1 ). 

SIOP: Sistema Integrado de Planejamento e Orçamento; PPA: Plano Plurianual.

 Painel de Planejamento Federal ( https://painelppa.economia.gov.br ). 

 Após a inclusão de todas as ações orçamentárias previstas nos Planos Nacionais, uma segunda etapa foi conduzida para verificar a necessidade de inclusão de outras ações orçamentárias relacionadas à agenda de SAN e que poderiam não estar presentes nos Planos Nacionais. Foi feita uma busca de termos relacionados à agenda de SAN nos campos do SIOP referentes aos programas, ações orçamentárias e objetivos. Estes termos foram definidos a partir das diretrizes da Política e do Plano Nacional de SAN, conforme regulamentação nacional ^14^ . Os termos utilizados foram: “Aliment”; “Nutric”; “Fome”; “Renda”; “Quilombo”; “Agrári”; “Agroecol”; “Agricult”; “Povo”; “Vulner”; “Indígen”; “Água”; “Soberania”; “SISAN”; “SESAN”; “PNSAN”; “Abastec”; “Aquicult”; “Hídric”; “PLANSAN”; “CONSEA”; “CAISAN”; “CNSAN”; “Comida”; “Cesta Básica”; “PNAE”; “PAA”; “PRONAF”; “SISVAN”; “Bolsa Família”; “Auxílio Brasil”; “Restaurante Popular”; “Cozinha Comunitária”; “Estoques Públicos”; “EPSAN”. As ações orçamentárias identificadas pela busca por termos foram analisadas por dois pesquisadores de forma independente, visando confirmar se possuíam relação com a agenda de SAN. As discordâncias na análise foram consensuadas entre os dois pesquisadores sobre a inclusão ou exclusão da ação orçamentária. Para a coleta, foi realizado o download dos dados abertos do SIOP para cada um dos anos analisados, e posteriormente, as ações orçamentárias selecionadas nas etapas anteriores tiveram sua dotação orçamentária e valor liquidado identificados coletados ( Figura 1 ). 

 As ações orçamentárias foram categorizadas conforme temas da agenda de SAN, considerando as oito diretrizes da PNSAN (14). Além destas, incorporou-se uma categoria relacionada à gestão do SISAN e da PNSAN (1). Uma ação orçamentária poderia ser incluída em mais de uma categoria, e o processo foi conduzido por dois pesquisadores de forma independente, que analisaram cada uma das ações orçamentárias e realizaram a categorização de forma subjetiva, considerando os detalhamentos das ações orçamentárias presentes no SIOP e descrições explicativas das diretrizes da PNSAN previamente elaboradas pelos pesquisadores ( Quadro 1 ). As discordâncias na categorização entre os pesquisadores foram consensuadas em reunião. Com o intuito de ilustrar as ações condizentes com cada categoria, o Quadro apresenta uma coluna com exemplos de ações orçamentárias localizadas a partir das etapas de categorização. 

**Quadro 1.** Categorias de classificação das ações orçamentárias relacionadas à agenda de Segurança Alimentar e Nutricional. Brasil, 2023.

**Categorias de segurança alimentar e nutricional**	**Conteúdo analisado nas ações orçamentárias com base na regulamentação nacional**	**Exemplos de ações orçamentárias (código Siop)**
1 - Acesso à Alimentação	Promoção do acesso universal à alimentação adequada e saudável, com prioridade para as famílias e pessoas em situação de insegurança alimentar e nutricional	Aquisição de Alimentos para Distribuição Gratuita (4244); Distribuição de Cestas de Alimentos a Famílias Carentes (2158); Assistência Financeira à relacionada ao Auxílio Emergencial da Emenda Constitucional nº 123, de 2022 (00UQ)
2 - Sistema Agroalimentar	Promoção do abastecimento e estruturação de sistemas sustentáveis e descentralizados, de base agroecológica, de produção, extração, processamento e distribuição de alimentos	Coordenação do Sistema de Assistência Técnica e Extensão Rural (2123); Desenvolvimento do Cooperativismo e Associativismo Rural (5696); Formação de Estoques Públicos (2130)
3 - Educação Alimentar e Nutricional e Pesquisa	Instituição de processos permanentes de educação alimentar e nutricional, pesquisa e formação nas áreas de segurança alimentar e nutricional e do direito humano à alimentação adequada	Estudos e Pesquisas Sobre Recuperação Nutricional e Alimentação Saudável (3890); Capacitação em Educação Alimentar, Nutricional e para o Consumo (2784); Apoio à Pesquisa e Desenvolvimento aplicados à Segurança Alimentar e Nutricional (0752)
4 - Povos e Comunidade Tradicionais e Populações Prioritárias	Promoção, universalização e coordenação das ações de segurança alimentar e nutricional voltadas para quilombolas e demais povos e comunidades tradicionais, povos indígenas e assentados da reforma agrária	Demarcação e Aviventação de Terras Indígenas (5004); Reconhecimento, Demarcação e Titulação de Áreas Remanescentes de Quilombos (1642); Apoio a Projetos de Segurança Alimentar e Nutricional dos Povos Indígenas (0B63)
5 - Ações de Saúde	Fortalecimento das ações de alimentação e nutrição em todos os níveis da atenção à saúde, de modo articulado às demais ações de segurança alimentar e nutricional	Análise de Perigos e Pontos Críticos de Controle de Contaminação em Indústrias de Alimentos (2120); Segurança Alimentar e Nutricional na Saúde (20QH); Distribuição de Micronutrientes para Crianças, Gestantes e Idosos em Áreas Endêmicas de Má Nutrição (4294)
6 - Acesso à Água	Promoção do acesso universal à água de qualidade e em quantidade suficiente, prioritário para as famílias em situação de insegurança hídrica e produção de alimentos da agricultura familiar, pesca e aquicultura	Estudos para Combate ao Desperdício de Água (3964); Construção e Ampliação Ou Melhoria dos Serviços de Abastecimento de Água para Controle de Agravos (3861); Fomento a Projetos de Manejo e Conservação de Recursos Hídricos (2957)
7 - Soberania Alimentar	Apoio a iniciativas de promoção da soberania alimentar, segurança alimentar e nutricional e do direito humano à alimentação adequada em âmbito internacional e a negociações internacionais	Relações e Negociações com a Organização das Nações Unidas para Agricultura e Alimentação (6100); Contribuição ao Fundo Índia-Brasil-África do Sul de Combate à fome (00ES); Contribuição ao Fundo da Agricultura Familiar do Mercosul (00ET)
8 - Monitoramento do Direito Humano à Alimentação Adequada	Monitoramento da realização do direito humano à alimentação adequada	Monitoramento da Situação Nutricional da População Brasileira (8519); Avaliação e monitoramento de Políticas de Desenvolvimento Social e Combate à Fome (4923); Ouvidoria Geral do Desenvolvimento Social e Combate à Fome (4907)
9 - Estrutura do Sistema Nacional de Segurança Alimentar e Nutricional	Estruturação e gestão do Sistema e da Política Nacional de Segurança Alimentar e Nutricional	Funcionamento do Conselho Nacional de Segurança Alimentar e Nutricional (4901); Consórcios de Segurança Alimentar e Desenvolvimento Local (8506); Apoio à Implantação e Gestão do Sistema Nacional de Segurança Alimentar e Nutricional (8624)

**Fonte:** baseado no Decreto nº 7.272, de 25 de agosto de 2010 (Brasil, 2010).

Siop: Sistema Integrado de Planejamento e Orçamento.

Para garantir a comparabilidade na análise do recurso investido, os montantes anuais foram deflacionados por meio do Índice de Preços ao Consumidor Amplo, medido pelo Instituto Brasileiro de Geografia e Estatística, considerando a inflação acumulada até janeiro de 2023.

 A partir dos dados coletados e corrigidos, foi conduzida uma análise descritiva com o objetivo de identificar as frequências absolutas e relativas do montante global de recurso de acordo com o tipo de alocação e a sua distribuição entre as categorias de SAN anteriormente apresentadas. Para a análise da série temporal, utilizou-se a regressão de Prais-Winsten como forma de mensurar a tendência, juntamente com o método de Cochrane-Orcutt para correção da autocorrelação seriada. A variação percentual anual (VPA) e os intervalos de confiança de 95% (IC95%) foram calculados por meio do ajuste da regressão ao logaritmo natural para a base 10 das proporções, tendo o ano como variável dependente. Adotou-se nível de significância de 5%. Valores de p não significantes (p ≥ 0,05) indicaram tendência de estabilidade; e valores de p significantes (p < 0,05), tendência crescente ou decrescente, conforme a variação anual positiva ou negativa, respectivamente ^15^ . 

Os dados coletados foram sistematizados e armazenados no programa Microsoft® Office Excel® 2010 e posteriormente tabulados e analisados pelo programa estatístico Stata 12.1 (College Station, Texas, Estados Unidos).

## RESULTADOS

Foram identificadas 349.009 ações orçamentárias relacionadas à agenda de SAN no período de 2000 a 2022. Quanto aos recursos previstos, a Tabela mostra que a dotação orçamentária (DO) do período foi R$ 4,6 trilhões, com variação anual positiva de 10,1%. A análise dos valores executados, representados pelo valor liquidado (VL) anual e no período, mostrou um montante de R$ 3,8 trilhões, correspondente a 83,74% da DO inicialmente alocada.

 A diferença entre a DO e o VL variou entre 71% e 92% ao longo do período. Os cinco anos com maior redução do VL em relação à DO mostraram uma variação entre 71% e 74%, sendo o menor valor aquele correspondente ao ano de 2013. Em contrapartida, os cinco anos com menores reduções mostraram variações entre 88% e 92%, com destaque positivo para o ano de 2022 (menor redução no VL) ( Tabela 1 ). 

 Em 2020 ocorreu a maior destinação de recursos na série temporal analisada, sendo que a DO e o VL acumulados entre 2020 e 2022 correspondem a quase 1/3 do montante global previsto e efetivamente executado em todo período analisado ( Tabela 1 ). 

**Tabela 1.** Distribuição e tendência do montante global de recursos públicos federais na área de Segurança Alimentar e Nutricional no período de 2000 a 2022, segundo tipo de alocação. Brasil, 2023.

**Ano**	**Dotação orçamentária**	**Valor liquidado**	**Diferença entre alocações**
	**Em bilhões R$**	**Em bilhões R$**	**%**
2000	42,51	36,51	85,88
2001	60,91	43,94	72,14
2002	61,79	45,97	74,39
2003	67,60	53,61	79,30
2004	86,90	70,85	81,53
2005	90,55	78,78	87,00
2006	104,78	92,60	88,37
2007	121,00	90,67	74,93
2008	138,65	104,11	75,08
2009	148,16	120,48	81,31
2010	161,72	129,96	80,36
2011	175,28	140,03	79,88
2012	227,51	166,04	72,97
2013	250,20	179,20	71,61
2014	245,16	189,46	77,28
2015	291,78	236,53	81,06
2016	257,53	226,96	88,13
2017	249,70	216,12	86,55
2018	241,02	220,84	91,62
2019	249,66	223,72	89,61
2020	685,04	601,62	87,82
2021	331,39	292,90	88,38
2022	336,53	312,52	92,86
**Total**	4.625,37	3.873,43	83,74
**Variação anual (%) (IC95%)** ^a^	10,1 (8,6; 11,7)	10,8 (9,6; 12)	-
**p-valor**	0,000	0,000	-
**Tendência**	Crescente	Crescente	-

IC95%: intervalo de confiança de 95%.

Nota: recursos deflacionados a preços de janeiro de 2023.

a Valores obtidos pela regressão de *Prais-Winsten* e calculados segundo a seguinte fórmula: variação anual =(-1+[10 ß)]x100, onde ß é o logaritmo de base natural resultante da regressão.

 Ao se analisar o comportamento do investimento nas categorias de SAN no período, observou-se diferenças no padrão de alocação dos recursos. Em ordem decrescente, as categorias “Ações de saúde”, “Soberania alimentar” e “Acesso à alimentação” apresentaram tendências crescentes da DO e do VL, sendo que as variações anuais da categoria “Ações de saúde” representaram mais de três vezes as variações do montante global. As categorias “Sistema alimentar” e “Monitoramento do DHAA” apresentaram uma tendência decrescente da DO e do VL. A categoria “Monitoramento do DHAA” recebeu destaque negativo nas variações anuais da DO e do VL, representando a maior diferença entre o valor previsto e o executado. As categorias “PCT e Pop. prioritárias” e “EAN e Pesquisa” sugeriram estabilidade para as variações anuais da DO e do VL durante o período analisado (p-valor ≥ 0,05). As categorias “Estrutura do Sisan” e “Acesso à água” também apresentaram tendência de estabilidade para a DO, no entanto, as variações anuais do VL para as mesmas categorias apresentaram uma tendência crescente e decrescente, respectivamente ( Gráfico 1 ). 

**Gráfico 1.**
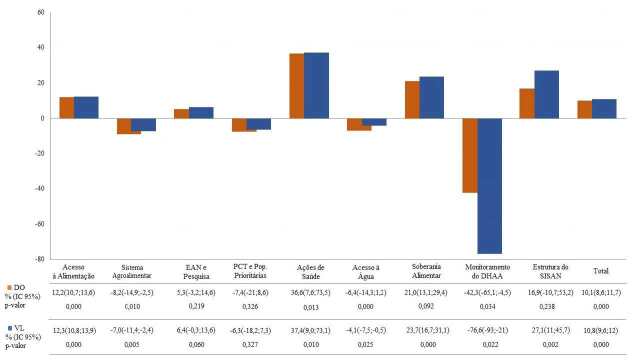
Variação da tendência de recursos federais no período de 2000 a 2022, segundo tipo de alocação e categorias de Segurança Alimentar e Nutricional. Brasil, 2023.

EAN: educação alimentar e nutricional; PCT: povos e comunidades tradicionais; DHAA: direito humano à alimentação adequada; Sisan: Sistema Nacional de Segurança Alimentar e Nutricional; IC95%: intervalo de confiança de 95%; DO: dotação orçamentária; VL: valor liquidado.

Nota: valores ajustados a preços de janeiro de 2023. Valores obtidos pela regressão de e calculados conforme fórmula: variação anual = (-1+[10 ß)]x100, onde ß é o logaritmo de base natural resultante da regressão.

 Quanto aos recursos executados, em média, 73,4% do montante total foi alocado na categoria “Acesso à alimentação”. Até 2014 as ações orçamentárias relacionadas a essa categoria não ultrapassaram 70% ao ano, diferente do que ocorreu nos anos seguintes, com destaque para o ano de 2020, onde a categoria totalizou 91% do valor executado. As ações orçamentárias vinculadas à categoria “Ações de saúde” começaram a ocupar uma maior fatia do VL a partir de 2008, mantendo-se relativamente estável até 2022 (entre 11,7% e 15,9%), com exceção do ano de 2020, quando representou apenas 5,5% do montante global. As ações orçamentárias relacionadas à “EAN e Pesquisa” chegaram a corresponder a 12% do valor total em 2014, antes de iniciarem um período persistente de queda e representarem apenas 3,4% em 2022, um percentual superior apenas ao período entre 2000 e 2005. As categorias “Sistema agroalimentar” e “Acesso à água” foram as que perderam maior representatividade no orçamento executado na agenda de SAN. As ações orçamentárias relacionadas ao “Sistema agroalimentar” ocupavam 36% do orçamento no ano 2000 e foram perdendo espaço de forma progressiva ao longo dos anos, ocupando apenas 2,3% do valor total em 2022. As ações orçamentárias de “Acesso à água” representavam 15,9% do orçamento executado em 2001, e em 2022 representaram apenas 0,2% ( Gráfico 2 ). 

**Gráfico 2.**
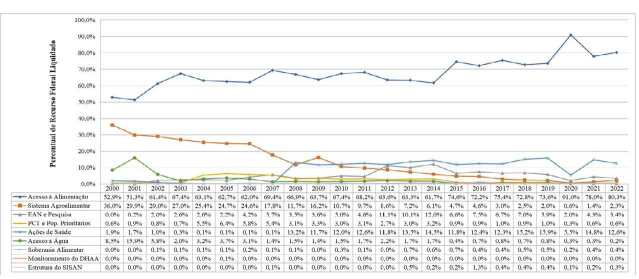
Percentuais dos valores liquidados na área de segurança alimentar e nutricional no período de 2000 a 2022, segundo a categoria de análise. Brasil, 2023.

EAN: educação alimentar e nutricional; PCT: povos e comunidades tradicionais; DHAA: direito humano à alimentação adequada; Sisan: Sistema Nacional de Segurança Alimentar e Nutricional.

Nota: valores ajustados a preços de janeiro de 2023.

## DISCUSSÃO

 Este estudo apresentou um panorama de duas décadas de financiamento federal para a agenda pública de SAN, tanto a partir de uma análise do montante global como por meio da análise do investimento público em áreas temáticas desta agenda. Os resultados encontrados no estudo refletem um processo histórico de construção da agenda de SAN no Brasil, e que envolve a compreensão de cenários políticos, socioeconômicos, demográficos e epidemiológicos, além de perpassar pelo contexto mundial e por acordos com organismos internacionais ^5^^,^^16^^,^^17^ . 

 O investimento federal brasileiro crescente em SAN a partir do início dos anos 2000 demonstra a priorização desta agenda no governo, tanto para atender às reivindicações sociais diante do cenário de fome no país nas décadas anteriores, quanto para cumprir com compromissos assumidos em acordos internacionais ^18, 19^ . O pioneirismo brasileiro no desenvolvimento de uma agenda pública de SAN que perpassou por programas de alimentação e nutrição e avançou para medidas de proteção social e transferência de renda, foram reconhecidos mundialmente por sua condução bem-sucedida e por seus expressivos resultados na redução IA grave entre 2000 a 2014 ^11^^,^^16^^,^^20^ . 

 A partir de 2016, inicia-se um período de decréscimo do montante global e uma menor diversificação do investimento nas diversas áreas de SAN, reflexo de uma mudança na condução econômica e do investimento nas políticas sociais. Assim como observado neste estudo, outra pesquisa que utilizou metodologia diferente de coleta e análise, também constatou cortes orçamentários de 76% nos recursos da área de SAN indicados no Plano Plurianual (PPA) de 2017, quando comparado ao plano de 2014 ^16^^,^^19^ . Este momento foi marcado por uma crise econômica internacional que afetou negativamente o preço das commodities, que historicamente movimentam a economia brasileira, somado a isso, o país passou por um processo de ruptura política e institucional, caracterizado entre outros elementos por um impeachment presidencial e a ascensão de uma agenda neoliberal e de austeridade fiscal. Presenciou-se um processo de desestruturação da agenda pública de SAN, com acentuados retrocessos potencializados em 2019, com a implementação de um Governo Federal pautado pelo negacionismo da situação de IA da população e na desresponsabilização do Estado na garantia da alimentação enquanto um direito constitucional ^17^ . 

 Outra oscilação constatada na análise deste estudo foi referente ao ano de 2020, com a maior destinação orçamentária anual, expressando quase o triplo de recurso investido no ano anterior. Este resultado estava relacionado com as ações do Governo Federal para a mitigação dos efeitos da covid-19, com a adoção de medidas emergenciais focadas no acesso à renda e aos alimentos ^8^^,^^17^ . No entanto é necessário considerar os efeitos do decréscimo de investimento nos anos anteriores e a redução na diversificação nas diversas áreas de SAN percebidas no estudo expressam resultados que corroboraram com a situação destacada em estudos prévios que retrataram o aumento da IA antes mesmo da pandemia ^16^^,^^17^ . 

 Vale destacar as diferenças percebidas entre os recursos indicados para a dotação orçamentária e o valor liquidado, tanto na análise do montante global, quanto das categorias de SAN. A diferença entre a dotação e a execução depende da capacidade de implementação das ações públicas. Além disso, a previsão de orçamento e a execução orçamentária para SAN são mediadas por inúmeros interesses e acordos, que envolvem diferentes poderes ^21^^–^^23^ . Como exemplo, estudos anteriores que analisaram o processo de governança de duas instâncias do poder executivo vinculadas à agenda pública de SAN, constataram que estes órgãos ainda possuem pouca articulação com o legislativo na consolidação desta área ^24^^,^^25^ . 

 O fato de o investimento público federal estar concentrado em ações relacionadas ao “Acesso à alimentação”, reforça uma agenda de SAN focada no assistencialismo e no desenvolvimento social, a partir da identificação de ações orçamentárias voltadas principalmente para programas de transferência direta de renda, auxílio emergencial e de distribuição de alimentos e refeições, situação já percebida por outros estudos ^21^^–^^23^ . Em países de renda média-baixa, como o Brasil, a associação direta entre a IA e a pobreza faz com o combate a esta condição seja incorporado principalmente a estratégias públicas de rompimento do ciclo da pobreza, acentuando o papel assistencial do Estado. Compreende-se que programas de acesso à alimentação buscam garantir um direito básico, numa lógica de proteção social a partir da oferta de serviços públicos ^26^ . Contudo, é necessário ponderar que centralizar a agenda de SAN em ações de acesso à alimentação limita o desenvolvimento de programas estruturantes que buscam garantir de forma contínua e concreta a garantia da SAN da população ^27^ . Fato este, também reconhecido em países de renda média-baixa, onde constatou-se que governos que favorecem ações assistenciais na agenda de SAN encontram limites no processo de governança e dificuldades na atenuação da IA ^28^^,^^29^ . 

 A priorização da categoria “Ações de saúde” na agenda de SAN corroborou com um estudo que analisou os recursos federais investidos em políticas da saúde num período equivalente, apresentando uma tendência crescente de 16,5% ^23^ . A implementação da agenda de SAN contou com a articulação intersetorial de ações de alimentação e nutrição inseridos no Sistema Único de Saúde ^19^ . Em estudo anterior que analisou a previsão e execução orçamentária da Política Nacional de Alimentação e Nutrição, entre 2003 a 2018, constatou-se que na maioria dos anos, tal execução ultrapassou 70% do recurso planejado. Analisou-se também, que a partir de 2006, o financiamento das ações de alimentação e nutrição foi progressivamente ampliado por meio de incentivo financeiro de custeio para apoiar a estruturação e a implementação das ações pelas secretarias estaduais e municipais de saúde ^30^ . 

 Os recursos executados nas categorias “Sistema agroalimentar” e “Acesso à água”, quando comparada com a tendência crescente do montante global, equiparam-se com um estudo que percebeu um aumento progressivo de recursos federais em políticas sociais, mas, de maneira setorial, as áreas de desenvolvimento agrário e saneamento, apresentaram uma tendência decrescente de -55,0% e -53,2%, respectivamente ^23^ . O que diz respeito ao decréscimo da categoria de “Sistema agroalimentar” equipara-se aos resultados de um estudo que identificou o quanto as políticas públicas de desenvolvimento agrário perderam investimento entre 2013 e 2019, com uma redução de R$ 624,1 milhões. Esta redução foi relacionada aos programas voltados para o fortalecimento da agricultura familiar e de aquisição de alimentos da agricultura familiar obtiveram redução de 30,9% e 67,1%, respectivamente ^23^ . A prioridade do governo brasileiro em relação à política agroalimentar é direcionada para um modelo de produção de alimentos pouco sustentável, baseado na monocultura, no uso de venenos e direcionado para o mercado externo. Esse modelo de sistema alimentar é causa da fome, da obesidade e das mudanças climáticas, pautado numa dinâmica de extração e exportação de recursos naturais, do agronegócio e do mercado de commodities ^17^ . 

 A categoria “Monitoramento do DHAA” apresentou a variação anual decrescente mais baixa e a maior diferença entre os tipos de alocação, apesar do monitoramento da situação de IA no Brasil ganhar força a partir de inquéritos de base populacional, com a aplicação de pesquisas públicas nacionais nos períodos de 2004, 2009, 2013 e 2017/2018 ^3^ . Destacando-se a necessidade maior investimento no aprimoramento dos mecanismos e na institucionalização do processo de monitoramento, como forma de atenuar os efeitos de crises financeiras e políticas apresentam nessa estratégia pública, expressos no caso do Brasil durante o período de 2019–2022 ^16^ . Torna-se importante registrar que o processo de monitoramento que ocorreu durante os anos de 2020–2022, foi conduzido e financiado por entidades não relacionadas à agenda pública de SAN, expressando um momento de crise da situação de IA nacional ^3^ . 

 Até o momento da realização do estudo, a agenda de SAN federal não possuía um fundo de financiamento próprio, de modo que o orçamento nesta área se encontrava dividido em diversos setores. Tal elemento tem sido visto como um grande desafio, destacado como um dos maiores entraves para a implementação e o desenvolvimento desta agenda pública ^20^^,^^21^^,^^24^^,^^25^ . O orçamento relacionado à essa agenda ainda apresenta grandes disputas, bem como os limites postos pelas restrições orçamentárias e pela distribuição desigual dos recursos entre os diferentes setores, o que repercute em limites para efetivar consolidação da política. Espera-se que, a partir de uma reorganização institucional da agenda de SAN, seja possível consolidar e garantir recurso federal na área a partir da sua inserção em instrumentos de gestão, tais como o plano nacional de SAN e o PPA federal ^24^^,^^25^ . 

 Apesar do recurso federal ter apresentado um caráter crescente ao longo do período analisado, não evitou os retrocessos na situação de IA ocorridos nos últimos anos. A partir de 2017, a IA atingiu cerca de 25,3 milhões de pessoas ^31^ . Dados recentes, do período de 2021 a 2022, indicaram que mais da metade (58,7%) da população brasileira estava em IA retratando um aumento progressivo desta condição no país ^3^ . A concentração de investimento em ações assistenciais na agenda de SAN, com o foco na garantia do acesso à alimentação, não se mostrou suficiente para atenuar de forma significativa a presença da IA na população ^28^^,^^29^ . 

O estudo não analisou o financiamento entre as diferentes gestões do Governo Federal, mas compreende-se que a destinação orçamentária está sujeita às prioridades estabelecidas em agendas de governo. Ressalta-se que o passo a passo metodológico utilizado mostra robustez e inovação para a área, podendo subsidiar e fomentar análises sequenciais com distintos níveis de interesse e profundidade.

## CONSIDERAÇÕES FINAIS

Os resultados do estudo demonstraram a necessidade de um investimento mais equânime entre as áreas de SAN, como forma de fortalecer políticas e programas públicas estruturantes, possibilitar a garantia da alimentação adequada enquanto direito humano, reduzir os níveis de IA e retirar novamente o Brasil do Mapa da Fome da ONU.

Corroborando, os últimos dados nacionais sobre IA reforçam a necessidade de ampliação do financiamento intersetorial para a agenda de SAN, visto que a concentração de aproximadamente 2/3 do orçamento para SAN exclusivamente destinado a ações de acesso à alimentação, não impediu a fome de retornar aos lares brasileiros.

Por se tratar de uma área que garante um direito constitucional, o orçamento público federal destinado à agenda de SAN não pode estar suscetível a crises políticas e econômicas, nem a interesses que divergem ou se afastam das reais necessidades da população brasileira.
