# Supplementary material for: Recurso público na agenda brasileira da Segurança Alimentar e Nutricional de 2000 a 2022
Source: Rev Saude Publica. 2024 Dec 20;58:54. [Article in Portuguese] doi: 10.11606/s1518-8787.2024058006104 (PMC11661774; doi:10.11606/s1518-8787.2024058006104)
Supplement: Translation

# Translation: Public resources on the Brazilian Food and Nutrition Security agenda from 2000 to 2022

**Published:** 2024-12-02

**Authors:** Milena Corrêa Martins, Mick Lennon Machado, Patrícia de Fragas Hinnig, Patrícia Maria de Oliveira Machado, Cristine Garcia Gabriel

**Affiliations:** I Universidade Federal de Santa Catarina. Centro de Ciências da Saúde. Programa de Pós-Graduação em Nutrição. Florianópolis, Santa Catarina, Brasil; II Universidade Federal de Santa Catarina. Centro de Ciências da Saúde. Teia de Articulação pelo Fortalecimento da Segurança Alimentar e Nutricional (TearSAN). Florianópolis, Santa Catarina, Brasil; III Universidade de Brasília. Faculdade de Ciências da Saúde. Departamento de Nutrição. Brasília, Distrito Federal, Brasil; IV Universidade Federal de Santa Catarina. Centro de Ciências da Saúde. Programa de Pós-Graduação em Saúde Coletiva. Florianópolis, Santa Catarina, Brasil

**Keywords:** Public Resources, Food and Nutrition Security, Time Series Studies

## Abstract

To analyze the time trend of federal public resources invested in the Brazilian Food and Nutrition Security agenda from 2000–2022.

A time series study was carried out with data collected from a publicly accessible system on the Federal Government’s planning and budget. We analyzed the budget actions and their resources indicated by the Budget Allocation and the Settled Amount. The actions were categorized into themes of the Food Nutrition Security agenda, analyzed using the Prais-Winsten regression, together with the Cochrane-Orcutt method. The values were adjusted by the Broad Consumer Price Index for January 2023.

Investment in the area of Food and Nutrition Security has shown an upward trend over two decades of federal funding, although it has shown fluctuations in certain years. The Budgetary Allocation showed an annual variation of 10.1%, and the Settled Amount obtained 10.8%. However, there is an imbalance in federal funding between strategic areas, with an upward trend in health (37.4%), food sovereignty (23.7%), and access to food (12.3%); and a downward trend in investment in some areas, such as agrarian development (-7.0%). The allocation of resources was concentrated on welfare actions to guarantee access to food, averaging 73.4% of the total resources spent.

The results show that public investment in Brazil’s Food and Nutrition Security agenda has been on the rise, although there have been fluctuations in certain years and a concentration of resources in specific areas. Despite having two thirds of the budget aimed exclusively at actions toward access to food, recent data shows that hunger still plagues the Brazilian reality. This reinforces the need for continuous and more equitable investment between areas, as a way of strengthening structuring public policies that permanently guarantee Food and Nutrition Security for the Brazilian population.

## INTRODUCTION

 Global food insecurity (FI) is increasing steadily, with around 900 million people in a situation of Severe FI by 2022 ^1^ . Projections by the International Food Policy Research Institute indicate that 65 million more people will be in this situation by 2030 ^2^ . In the same direction as the global data, 33.1 million people were in severe FI in Brazil, from 2021 to 2022 ^3^ . 

 This scenario has mobilized different countries and organizations to formulate and allocate resources to the Food and Nutrition Security (FNS) agenda ^4^ . Some national strategies stand out, which are generally intersectoral in nature, allocating resources to various areas of FNS, such as: health, education, social protection, agriculture, the environment, among others ^5, 6^ . During the COVID-19 pandemic, these strategies have seen an increase in resources due to the rise in FI ^7^^,^^8^ . 

 The first measures adopted by 54 countries in response to the pandemic involved 496 actions and a public investment of US$ 47.6 billion related to FNS ^9^ . In 2022, the World Bank, as part of a global response to the FI crisis, made 30 million dollars available to finance projects related to the area ^10^ . 

 Brazil has intensified its actions to fight hunger since the early 2000s, with emphasis on the publication of the Organic Law on Food and Nutrition Security in 2006, which institutionalized a public agenda organized intersectorally in the country via the National Food and Nutrition Security System (SISAN), responsible for removing Brazil from the Hunger Map in 2014 ^11^ . According to the Hunger and Nutrition Commitment Index (HANCI), a global index that assesses national political commitment to fighting hunger and FI considering financial variables, in 2019 Brazil ranked second out of forty-five lower-middle-income countries analyzed ^12^ . 

 In Brazil’s institutional logic, the public budget is the instrument used to plan the use of the money collected from taxes. Its analysis makes it possible to understand government choices and the trajectory of policies, revealing how much is spent and the priority agendas. In this sense, analyses that enable comparisons over time provide information on the characteristics of government agendas, the priority agendas and the actions taken to implement the FNS agenda ^13^ . Considering the paucity of research in the area, this study sought to analyze the temporal trend of federal public resources invested in the Brazilian FNS agenda over the last two decades (2000 and 2022), a period marked by significant advances, but also by substantial setbacks in the area. 

## METHODS

 This is a time series study of the amount of federal public funds invested in the area of FNS between 2000 and 2022. The data was collected from the *Sistema Integrado de Planejamento e Orçamento* (SIOP – Integrated Planning and Budget System) ( https://www.siop.planejamento.gov.br ), a publicly accessible system that gathers information on the federal government’s planning and budget. The budget actions were analyzed, and their resources indicated by the budget allocation and the amount settled. The budget allocation refers to the amount planned and approved in the public budget, while the settled amount shows what was actually executed in the budget. 

Given the complexity and multidimensionality of the FNS agenda, this study faced the methodological challenge of determining which budget actions would be included in the analysis. We opted for broader inclusion criteria, thus ensuring the analysis of budget actions associated with the guidelines of the National Food and Nutrition Security Policy (PNSAN), without restricting ourselves exclusively to those linked to or originating directly from this public policy. This theoretical-methodological approach is based on the understanding that the intersectoral nature of PNSAN implies the allocation and use of public resources linked to different policies for the effective realization of the FNS agenda in the country. This understanding is also reflected in PNSAN’s main planning and management instrument, the National FNS Plans, which describe budgetary actions linked to various policies in order to achieve its goals.

 For this reason, the identification of budget actions associated with the FNS agenda was carried out in two stages, based on a protocol that had previously been constructed and agreed between the researchers ( Figure 1 ). Initially, the budget actions were defined based on the goals of the National FNS Plans. Four documents were analyzed: the first version of the plan (2012-2015 period), the second version (2016-2019 period), as well as their respective revisions. As these documents present the budget actions differently, a flowchart was established to standardize data collection ( Figure 1 ). 

**Figure 1.** Protocol for identifying budget actions related to the Food and Nutrition Security agenda in the Integrated Planning and Budgeting System. Brazil, 2023.

SIOP: Integrated Planning and Budgeting System; PPA: Multiannual Plan.

 Federal Planning Panel ( https://painelppa.economia.gov.br ). 

 Once all the budget actions provided for in the National Plans were included, a second stage was carried out to check for the need to include other budget actions related to the FNS agenda that might not have been included in the National Plans. A search was made for terms related to the FNS agenda in the SIOP fields referring to programs, budget actions and objectives. These terms were defined based on the guidelines of the National FNS Policy and Plan, in accordance with national regulations ^14^ . The terms used were: “Aliment”; “Nutric”; “Fome”; “Renda”; “Quilombo”; “Agrári”; “Agroecol”; “Agricult”; “Povo”; “Vulner”; “Indígen”; “Água”; “Soberania”; “SISAN”; “SESAN”; “PNSAN”; “Abastec”; “Aquicult”; “Hídric”; “PLANSAN”; “CONSEA”; “CAISAN”; “CNSAN”; “Comida”; “Cesta Básica”; “PNAE”; “PAA”; “PRONAF”; “SISVAN”; “Bolsa Família”; “Auxílio Brasil”; “Restaurante Popular”; “Cozinha Comunitária”; “Estoques Públicos”; “EPSAN”. The budget actions identified by the search for terms were analyzed by two researchers independently, in order to confirm whether they were related to the FNS agenda. Disagreements in the analysis were settled between the two researchers as to whether the budget action should be included or excluded. For the collection, open data was downloaded from SIOP for each of the years analyzed, and then the budget actions selected in the previous steps had their budget allocation and settled amount identified collected ( Graph 1 ). 

 Budgetary actions were categorized according to themes on the FNS agenda, taking into account the eight guidelines of PNSAN (14). In addition, a category related to the management of SISAN and PNSAN was incorporated ( Chart 1 ). A budget action could be included in more than one category, and the process was conducted independently by two researchers, who analyzed each of the budget actions and carried out the categorization subjectively, considering the details of the budget actions in the SIOP and explanatory descriptions of the PNSAN guidelines previously prepared by the researchers ( Chart 1 ). Disagreements in the categorization between the researchers were agreed upon in a meeting. In order to illustrate the actions consistent with each category, the table shows a column with examples of budget actions located based on the categorization stages. 

**Chart 1.** Classification categories of budget actions related to the Food and Nutrition Security agenda. Brazil, 2023.

**Categories of food and nutrition security**	**Content analyzed in budget actions based on national regulations**	**Examples of budget actions (SIOP code** )
1 - Access to Food	Promoting universal access to adequate and healthy food, with priority for families and people in situations of food and nutritional insecurity	Acquisition of Food for Free Distribution (4244); Distribution of Food Baskets to Needy Families (2158); Financial Assistance related to Emergency Aid under Constitutional Amendment No. 123 of 2022 (00UQ)
2 - Agri-food system	Promoting the supply and structuring of sustainable and decentralized agro-ecological food production, extraction, processing, and distribution systems	Coordination of the Technical Assistance and Rural Extension System (2123); Development of Cooperatives and Rural Associations (5696); Formation of Public Stocks (2130)
3 - Food and Nutrition Education and Research	Establishment of permanent food and nutrition education, research, and training processes in the areas of food and nutrition security and the human right to adequate food	Studies and Research on Nutritional Recovery and Healthy Eating (3890); Training in Food, Nutrition and Consumer Education (2784); Support for Research and Development applied to Food and Nutrition Security (0752)
4 - Traditional Peoples and Communities and Priority Populations	Promotion, universalization and coordination of food and nutritional security actions aimed at *quilombolas* and other traditional peoples and communities, Indigenous peoples, and agrarian reform settlers	Demarcation and Revival of Indigenous Lands (5004); Recognition, Demarcation and Titling of Areas Remaining from Quilombos (1642); Support for Food and Nutritional Security Projects for Indigenous Peoples (0B63)
5 - Health actions	Strengthening food and nutrition actions at all levels of health care, in conjunction with other food and nutrition security actions	Hazard Analysis and Critical Control Points for Contamination in Food Industries (2120); Food and Nutrition Security in Health (20QH); Distribution of Micronutrients to Children, Pregnant Women, and Older Adults in Areas Endemic for Malnutrition (4294)
6 - Access to Water	Promoting universal access to quality water in sufficient quantity, as a priority for families in situations of water insecurity and food production in family farming, fishing, and aquaculture.	Studies to Combat Water Waste (3964); Construction and Expansion or Improvement of Water Supply Services to Control Diseases (3861); Promotion of Water Resource Management and Conservation Projects (2957)
7 - Food Sovereignty	Support for initiatives to promote food sovereignty, food and nutrition security and the human right to adequate food at international level and international negotiations	Relations and Negotiations with the Food and Agriculture Organization of the United Nations (6100); Contribution to the India-Brazil-South Africa Fund to Fight Hunger (00ES); Contribution to the Mercosur Family Farming Fund (00ET)
8 - Monitoring the Human Right to Adequate Food	Monitoring the realization of the human right to adequate food	Monitoring the Nutritional Situation of the Brazilian Population (8519); Evaluation and Monitoring of Social Development and Fight against Hunger Policies (4923); General Ombudsman for Social Development and Fight against Hunger (4907)
9 - Structure of the National Food and Nutrition Security System	Structuring and managing the National Food and Nutrition Security Policy and System	Operation of the National Food and Nutrition Security Council (4901); Food Security and Local Development Consortia (8506); Support for the Implementation and Management of the National Food and Nutrition Security System (8624)

**Source:** based on Decree No. 7.272 of August 25, 2010 (Brazil, 2010).

SIOP: Integrated Planning and Budgeting System.

To ensure comparability in the analysis of the resources invested, the annual amounts were deflated using the Broad Consumer Price Index, measured by the Brazilian Institute of Geography and Statistics, considering accumulated inflation until January 2023.

 Based on the data collected and corrected, a descriptive analysis was carried out in order to identify the absolute and relative frequencies of the overall amount of resources according to the type of allocation and their distribution among the FNS categories presented above. To analyze the time series, Prais-Winsten regression was used to measure the trend, along with the Cochrane-Orcutt method to correct for serial autocorrelation. The annual percentage change (APC) and 95% confidence intervals (95%CI) were calculated by adjusting the regression to the natural logarithm to the base 10 of the proportions, with the year as the dependent variable. A significance level of 5% was adopted. Non-significant p-values (p ≥ 0.05) indicated a stable trend; and significant p-values (p < 0.05), an increasing or decreasing trend, depending on the positive or negative annual variation, respectively ^15^ . 

The data collected was systematized and stored in the Microsoft® Office Excel® 2010 program and then tabulated and analyzed using the Stata 12.1 statistical program (College Station, Texas, United States).

## RESULTS

A total of 349,009 budget actions related to the FNS agenda were identified for the period 2000 to 2022. The table shows that the budget allocation (BA) for the period was R$4.6 trillion, with a positive annual variation of 10.1%. Analysis of the amounts executed, represented by the annual settled amount (SA) and in the period, showed an amount of R$3.8 trillion, corresponding to 83.74% of the initially allocated BA.

 The difference between the BA and the SA varied between 71% and 92% over the period. The five years with the greatest reduction in the SA in relation to the BA showed a variation between 71% and 74%, with the lowest value corresponding to 2013. On the other hand, the five years with the smallest reductions showed variations between 88% and 92%, with a positive highlight for the year 2022 (the smallest reduction in the SA) ( Table 1 ). 

 2020 saw the largest allocation of resources in the time series analyzed, with the accumulated BA and SA from 2020 to 2022 corresponding to almost 1/3 of the overall amount planned and actually executed in the entire period analyzed ( Table 1 ). 

**Table 1.** Distribution and trend of the overall amount of federal public resources in the area of Food and Nutrition Security from 2000 to 2022, by type of allocation. Brazil, 2023.

**Ano**	**Budget allocation**	**Settled amount**	**Difference between allocations**
	**In billion R$**	**In billion R$**	**%**
2000	42.51	36.51	85.88
2001	60.91	43.94	72.14
2002	61.79	45.97	74.39
2003	67.60	53.61	79.30
2004	86.90	70.85	81.53
2005	90.55	78.78	87.00
2006	104.78	92.60	88.37
2007	121.00	90.67	74.93
2008	138.65	104.11	75.08
2009	148.16	120.48	81.31
2010	161.72	129.96	80.36
2011	175.28	140.03	79.88
2012	227.51	166.04	72.97
2013	250.20	179.20	71.61
2014	245.16	189.46	77.28
2015	291.78	236.53	81.06
2016	257.53	226.96	88.13
2017	249.70	216.12	86.55
2018	241.02	220.84	91.62
2019	249.66	223.72	89.61
2020	685.04	601.62	87.82
2021	331.39	292.90	88.38
2022	336.53	312.52	92.86
**Total**	4.625.37	3.873.43	83.74
**Annual variation (%) (95%IC)** ^a^	10.1 (8.6; 11.7)	10.8 (9.6; 12)	-
**p-value**	0,000	0,000	-
**Trend**	Increasing	Decreasing	-

95%CI: 95% confidence interval.

Note: resources deflated at January 2023 prices.

a Values obtained by Prais-Winsten regression and calculated according to the following formula: annual variation =(-1+[10 ß)]x100, where ß is the natural base logarithm resulting from the regression.

 When analyzing the behavior of investment in FNS categories over the period, differences were observed in the pattern of resource allocation. In descending order, the categories “Health actions,” “Food sovereignty,” and “Access to food” showed increasing trends in the BA and the SA, with the annual variations in the “Health actions” category representing more than three times the variations in the overall amount. The categories “Food system” and “Monitoring the HRAF” showed a downward trend in BA and SA. The “Monitoring the HRAF” category had a negative impact on the annual variations in the BA and the SA, representing the biggest difference between the amount planned and the amount executed. The categories “TPC and Priority Population” and “FNE and Research” suggested stability in the annual variations in BA and SA during the period analyzed (p-value ≥ 0.05). The categories “Structure of Sisan” and “Access to water” also showed a stable trend for the BA, however, the annual variations in the SA for the same categories showed an increasing and decreasing trend, respectively ( Graph 1 ). 

**Graph 1.** Variation in the trend of federal resources from 2000 to 2022, by type of allocation and Food and Nutrition Security categories. Brazil, 2023.

FNE: food and nutrition education; TPC: traditional peoples and communities; HRAF: human right to adequate food; SISAN: National Food and Nutrition Security System; 95%CI: 95% confidence interval; BA: budget allocation; SA: settled amount.

Note: values adjusted to January 2023 prices. Values obtained by Prais-Winsten regression and calculated according to the formula: annual variation = (-1+[10 ß)]x100, where ß is the natural base logarithm resulting from the regression.>

 On average, 73.4% of the total amount was allocated to the “Access to food” category. Until 2014, budget actions related to this category did not exceed 70% per year, unlike what happened in the following years, especially in 2020, when the category accounted for 91% of the amount executed. Budgetary actions linked to the “Health actions” category began to occupy a larger share of the SA from 2008 onwards, remaining relatively stable until 2022 (from 11.7% to 15.9%), with the exception of 2020, when it accounted for only 5.5% of the overall amount. Budget actions related to “FNE and Research” reached 12% of the total amount in 2014, before beginning a persistent period of decline and representing just 3.4% in 2022, a percentage higher only than the 2000-2005 period. The categories “Agri-food system” and “Access to water” were the ones that lost the most representation in the budget executed for the FNS agenda. Budgetary actions related to the “Agri-food system” accounted for 36% of the budget in 2000 and progressively lost ground over the years, accounting for only 2.3% of the total amount in 2022. The “Access to water” budget actions accounted for 15.9% of the executed budget in 2001, and in 2022 only 0.2% ( Graph 2 ). 

**Graph 2.** Percentages of the amounts settled in the area of food and nutrition security in the 2000-2002 period, according to the category of analysis. Brazil, 2023.

FNE: food and nutrition education; TPC: traditional peoples and communities; HRAF: human right to adequate food; SISAN: National Food and Nutrition Security System.

Note: values adjusted to January 2023 prices.

## DISCUSSÃO

 This study presented an overview of two decades of federal funding for the public FNS agenda, both through an analysis of the overall amount and through an analysis of public investment in thematic areas of this agenda. The results found in the study reflect a historical process of building the FNS agenda in Brazil, which involves understanding political, socioeconomic, demographic, and epidemiological scenarios, as well as the global context and agreements with international organizations ^5^^,^^16^^,^^17^ . 

 Brazil’s growing federal investment in FNS from the early 2000s demonstrates the government’s prioritization of this agenda, both to meet social demands in the face of the country’s hunger scenario in previous decades, and to comply with commitments made in international agreements ^2019^^,^^19^ . Brazil’s pioneering role in the development of a public FNS agenda, which included food and nutrition programs and progressed to social protection and income transfer measures, has been recognized worldwide for its successful conduct and its significant results in reducing severe hunger between 2000 and 2014 ^11^^,^^16^^,^^20^ . 

 From 2016 onwards, there was a period of decrease in the overall amount and less diversification of investment in the various areas of FNS, reflecting a change in economic management and investment in social policies. As observed in this study, another study that used a different collection and analysis methodology also found budget cuts of 76% in the FNS resources indicated in the 2017 Pluriannual Plan (PPA), as compared to the 2014 plan ^16^^,^^19^ . This moment was marked by an international economic crisis that negatively affected the price of commodities, which historically drive the Brazilian economy. In addition, the country went through a process of political and institutional rupture, characterized among other elements by presidential impeachment and the rise of a neoliberal and fiscal austerity agenda. A process of dismantling the public FNS agenda was witnessed, with sharp setbacks potentialized in 2019, with the implementation of a Federal Government guided by denial of the population’s FI situation and the state’s lack of responsibility in guaranteeing food as a constitutional right ^17^ . 

 Another fluctuation found in the analysis of this study was in 2020, with the highest annual budget allocation, expressing almost triple the resources invested in the previous year. This result was related to the federal government’s actions to mitigate the effects of COVID-19, with the adoption of emergency measures focused on access to income and food ^8^^,^^17^ . However, it is necessary to consider that the effects of the decrease in investment in previous years and the reduction in diversification in the various areas of FNS perceived in the study express results that corroborate the situation highlighted in previous studies, which portrayed the increase in FI even before the pandemic ^16^^,^^17^ . 

 It is worth noting the differences between the resources indicated in the budget allocation and the amount paid out, both when analyzing the overall amount and the FNS categories. The difference between allocation and execution depends on the capacity to implement public actions. In addition, budget forecasting and budget execution for FNS are mediated by numerous interests and agreements involving different powersd ^21^ - ^23^ . As an example, previous studies that analyzed the governance process of two instances of the executive branch linked to the public FNS agenda found that these bodies still have little articulation with the legislative branch in the consolidation of this area ^24^^,^^25^ . 

 The fact that federal public investment is concentrated on actions related to “access to food” reinforces a FNS agenda focused on welfare and social development, based on the identification of budgetary actions aimed mainly at direct income transfer programs, emergency aid and food and meal distribution, a situation already noted by other studies ^21–23^ . In lower-middle-income countries like Brazil, the direct association between FI and poverty means that the fight against this condition is mainly incorporated into public strategies to break the cycle of poverty, accentuating the state’s welfare role. It is understood that food access programs seek to guarantee a basic right, in a logic of social protection based on the provision of public services ^26^ . However, it is necessary to consider that centralizing the FNS agenda on actions to provide access to food limits the development of structuring programs that seek to guarantee the FNS of the population in a continuous and concrete way ^27^ . This fact has also been recognized in lower-middle-income countries, where it has been found that governments that favor welfare actions in the FNS agenda encounter limits in the governance process and difficulties in mitigating FI ^28^^,^^29^ . 

 The prioritization of the “health actions” category in the FNS agenda corroborated a study that analyzed the federal resources invested in health policies in an equivalent period, showing an upward trend of 16.5% ^23^ . The implementation of the FNS agenda relied on the intersectoral articulation of food and nutrition actions within the Brazilian Unified Health System ^19^ . In a previous study that analyzed the budget forecast and execution of the National Food and Nutrition Policy from 2003 to 2018, it was found that in most years, this execution exceeded 70% of the planned resource. It was also analyzed that from 2006 onwards, the financing of food and nutrition actions was progressively expanded through financial incentives to support the structuring and implementation of actions by state and municipal health departments ^30^ . 

 The resources invested in the “Agri-food system” and “Access to water” categories, when compared to the growing trend in the overall amount, are in line with a study that found a progressive increase in federal resources in social policies, but, in sectoral terms, the areas of agrarian development and sanitation showed a downward trend of -55.0% and -53.2%, respectively ^23^ . The decrease in the “Agri-food system” category is in line with the results of a study that identified how much public policies for agrarian development lost in investment from 2013 to 2019, with a reduction of R$624.1 million. This reduction was related to programs aimed at strengthening family farming and the acquisition of food from family farming, which saw a reduction of 30.9% and 67.1%, respectively ^23^ . The Brazilian government’s agri-food policy priority is geared towards an unsustainable model of food production, based on monoculture and use of pesticides and aimed at the foreign market. This model of food system causes hunger, obesity, and promotes climate change; it serves a dynamic of extraction and exportation of natural resources, the agribusiness, and the commodities market ^17^ . 

 The “Monitoring the HRAF” category showed the lowest decreasing annual variation and the greatest difference between the types of allocation, even though the monitoring of the FI situation in Brazil has gained strength from population-based surveys, with the application of national public surveys in the periods 2004, 2009, 2013, and 2017/2018 ^3^ . This highlights the need for greater investment in improving the mechanisms and institutionalization of the monitoring process, as a way of mitigating the effects of financial and political crises on this public strategy, expressed in the case of Brazil during the 2019-2022 period ^16^ . It is important to note that the monitoring process that took place during the years 2020-2022 was conducted and financed by entities unrelated to the FNS public agenda, expressing a moment of crisis in the national FI situation ^3^ . 

 Up until the time of the study, the federal FNS agenda did not have its own funding fund, so the budget in this area was divided between various sectors. This element has been seen as a major challenge, highlighted as one of the biggest obstacles to the implementation and development of this public agenda ^20^^,^^21^^,^^24^^,^^25^ . The budget related to this agenda is still subject to major disputes, as well as the limits imposed by budget restrictions and the unequal distribution of resources between the different sectors, which has repercussions on the consolidation of the policy. It is hoped that, with an institutional reorganization of the FNS agenda, it will be possible to consolidate and guarantee federal resources in the area by including them in management instruments, such as the national FNS plan and the federal PPA ^24^^,^^25^ . 

 Although federal funding has been increasing over the period analyzed, it has not prevented the backsliding in the FI situation that has occurred in recent years. As of 2017, FI affected around 25.3 million people ^31^ . Recent data, from 2021 to 2022, indicated that more than half (58.7%) of the Brazilian population was in FI, portraying a progressive increase in this condition in the country ^3^ . The concentration of investment in assistance actions on the FNS agenda, with a focus on guaranteeing access to food, has not proved sufficient to significantly mitigate the presence of FI in the population ^28^^,^^29^ . 

The study did not analyze funding across the different administrations of the federal government, but it is understood that budget allocation is subject to the priorities established in government agendas. It should be noted that the methodological step-by-step used shows robustness and innovation for the area and can provide support and foster sequential analyses with different levels of interest and depth.

## FINAL CONSIDERATIONS

The results of the study showed the need for more equitable investment between FNS areas, as a way of strengthening structuring public policies and programs, making it possible to guarantee adequate food as a human right, reduce FI levels and remove Brazil from the UN Hunger Map once again.

Corroborating this, the latest national data on FI reinforces the need to expand intersectoral funding for the FNS agenda, given that the concentration of approximately 2/3 of the FNS budget exclusively for food access actions has not prevented hunger from returning to Brazilian homes.

As this is an area that guarantees a constitutional right, the federal public budget earmarked for the FNS agenda cannot be susceptible to political and economic crises, nor to interests that diverge or move away from the real needs of the Brazilian population.
